# Efficacy and Safety of 10 kHz Spinal Cord Stimulation for the Treatment of Chronic Pain: A Systematic Review and Narrative Synthesis of Real-World Retrospective Studies

**DOI:** 10.3390/biomedicines9020180

**Published:** 2021-02-11

**Authors:** Ganesan Baranidharan, Deborah Edgar, Beatrice Bretherton, Tracey Crowther, Abdul-Ghaaliq Lalkhen, Ann-Katrin Fritz, Girish Vajramani

**Affiliations:** 1 Leeds Teaching Hospitals NHS Trust Leeds, Leeds LS1 3EX, UK; beatrice.bretherton@nhs.net (B.B.); tracey.crowther1@nhs.net (T.C.); 2 School of Medicine, Faculty of Medicine and Health, University of Leeds, Leeds LS2 9JT, UK; 3 Commexus Ltd., Dunblane FK15 0DF, UK; dedgar@commexus.com; 4 School of Biomedical Sciences, Faculty of Biological Sciences, University of Leeds, Leeds LS2 9JT, UK; 5 Manchester Center for Clinical Neurosciences, Salford M6 8HD, UK; abdul.lalkhen@srft.nhs.uk; 6 Pain Management Centre, Norfolk and Norwich University Hospital, Norwich NR4 7UY, UK; ann-katrin.fritz@nnuh.nhs.uk; 7 Centre for Functional Neurosurgery, University Hospital Southampton NHS Foundation Trust, Hampshire SO16 6YD, UK; girish.vajramani@nhs.net

**Keywords:** 10 kHz SCS, chronic pain, observational studies, real-world studies

## Abstract

10 kHz spinal cord stimulation (SCS) is increasingly utilized globally to treat chronic pain syndromes. Real-world evidence complementing randomized controlled trials supporting its use, has accumulated over the last decade. This systematic review aims to summarize the retrospective literature with reference to the efficacy and safety of 10 kHz SCS. We performed a systematic literature search of PubMed between 1 January 2009 and 21 August 2020 for English-language retrospective studies of ≥3 human subjects implanted with a Senza^®^ 10 kHz SCS system and followed-up for ≥3 months. Two independent reviewers screened titles/abstracts of 327 studies and 46 full-text manuscripts. In total, 16 articles were eligible for inclusion; 15 reported effectiveness outcomes and 11 presented safety outcomes. Follow-up duration ranged from 6–34 months. Mean pain relief was >50% in most studies, regardless of follow-up duration. Responder rates ranged from 67–100% at ≤12 months follow-up, and from 46–76% thereafter. 32–71% of patients decreased opioid or nonopioid analgesia intake. Complication incidence rates were consistent with other published SCS literature. Findings suggest 10 kHz SCS provides safe and durable pain relief in pragmatic populations of chronic pain patients. Furthermore, it may decrease opioid requirements, highlighting the key role 10 kHz SCS can play in the medium-term management of chronic pain.

## 1. Introduction

Chronic pain is reported to affect around 20% of adults [1,2] and is a significant cause of suffering and disability [3]. The socioeconomic burden is substantial in terms of health care expenditure [4,5,6,7] and reduced work productivity [2,8,9,10]. A multidisciplinary approach to chronic pain treatment may include pharmacotherapy, psychological and physical therapy, neuromodulation, nerve ablation and therapeutic injections, and nerve stimulation [11]. Despite the numerous treatments available, unrelieved pain is highly prevalent [12]. Increasing reliance on opioids to treat chronic pain has become problematic in developed countries [13,14]. While short-term opiate use appears to be beneficial in chronic pain [15], long-term use has little supportive evidence, carries the risk of addiction [16,17,18,19] and is associated with common adverse effects such as constipation, nausea, and drowsiness. Less-known effects have also been reported, including opioid-induced hyperalgesia, muscle rigidity, hormonal and immunological imbalances, as well as severe but infrequent issues such as respiratory depression [20,21,22]. The prevalence of opioid misuse and dependence is reported to range up to 32% [16,23,24]. Alternative pain relief treatments for chronic pain that avoid the risks associated with long-term opioid therapy and the negative side-effects of anti-epileptic medications are needed.

For nearly a decade, 10 kHz spinal cord stimulation (SCS) has been used to treat chronic pain syndromes of various etiologies. This minimally invasive therapy delivers high-frequency electrical pulses to the spinal cord without producing paresthesia [25]. A high quality randomized controlled trial (RCT) established the therapy as superior to traditional low-frequency SCS (LF-SCS) in patients with chronic back and leg pain [25,26]. Other prospective case series found similar benefits in patients with predominant axial back pain [27,28] and nonsurgical back pain [29,30]. The positive outcomes from these studies prompted investigators to prospectively explore other possible indications, including chronic postsurgical pain [31], pelvic pain [32], painful diabetic neuropathy [33], abdominal pain [34], migraine [35], and neck and/or upper limb pain [36], with encouraging results.

It is accepted that robustly designed RCTs are the reference standard for evaluating the causal relationships between a treatment and an outcome of interest (as far as this is possible) whilst minimizing bias. Controlling for patient selection and implementing rigorous clinical protocols in an RCT evaluates treatment outcomes in ideal circumstances but may not be a true reflection of how the treatment will work in a non-trial setting [37,38,39,40]. Furthermore, it is not always possible or realistic to implement an RCT for practical, economic, or ethical reasons. In this scenario, prospective observational studies are often a pragmatic design choice, but the administrative requirements are still a significant undertaking, and patient selection criteria can be challenging to implement. Retrospective research offers the opportunity to harness valuable insights from real-world clinical experience already captured in medical records. Interpreted carefully, and bearing in mind their limitations, retrospective studies serve a useful purpose as complementary evidence to RCTs [39]. Up to now, several literature reviews have summarized clinical outcomes from 10 kHz SCS studies conducted over the last decade [41,42,43,44]. The reviews were either very broad, including both prospective and retrospective studies [41,42], or focused on a single indication [44] or outcome [43]. None of the literature reviews were conducted systematically or examined safety outcomes in detail across all included studies.

Currently, a comprehensive overview of reported clinical experience, including both effectiveness and safety outcomes, is lacking. Considering the importance of real-world evidence in clinical decision making, there is an unmet need for a literature review specifically summarizing retrospective real-world studies from clinical practice. Therefore, to gain more insight into the clinical benefits and safety profile of 10 kHz SCS in real-world conditions, we performed a systematic review of all available retrospective studies.

## 2. Methods

### 2.1. Literature Search

The literature search was conducted using the National Library of Medicine PubMed electronic database. Combinations of relevant keywords were used, including spinal cord stimulation, 10 kHz, HF10, high-frequency, and kilohertz frequency. Since the Senza^®^ 10 kHz SCS system received its first regulatory approval in 2010 (Nevro Corp., Redwood City, CA, USA), the literature was searched from 1 January 2009. The search was executed on 21 August 2020.

### 2.2. Study Eligibility

Studies were included if the clinical outcome or safety data were collected retrospectively from at least three human subjects implanted with a Senza^®^ 10 kHz SCS system. The minimum follow-up period was 3 months to exclude studies focused on temporary trial or very short-term outcomes. Peripheral nerve stimulation applications were excluded. Only English-language articles published in peer-reviewed journals were considered.

### 2.3. Reviewers

Two reviewers worked independently to screen titles and abstracts for original studies that met the eligibility criteria (with full-text manuscripts consulted for verification if necessary). Disagreements were resolved by consensus. Full-text manuscripts were obtained for all studies determined to be eligible.

### 2.4. Data Extraction

Data were extracted from all eligible studies by the principal reviewer, tabulated in a Microsoft Excel spreadsheet, and crosschecked by the second reviewer. Study design variables were documented, along with the primary outcomes of interest, including average pain relief of the cohort versus baseline, the group responder rate (with response defined as ≥50% pain relief from baseline), medication change, functional capacity and quality of life (QoL) outcomes, and the incidence and sequelae of complications. The heterogeneity of patient populations in the studies precluded traditional meta-analytic approaches. Instead, we provide a narrative synthesis of study key features and the principal outcomes of interest.

## 3. Results

### 3.1. Study Selection

**Figure 1:**
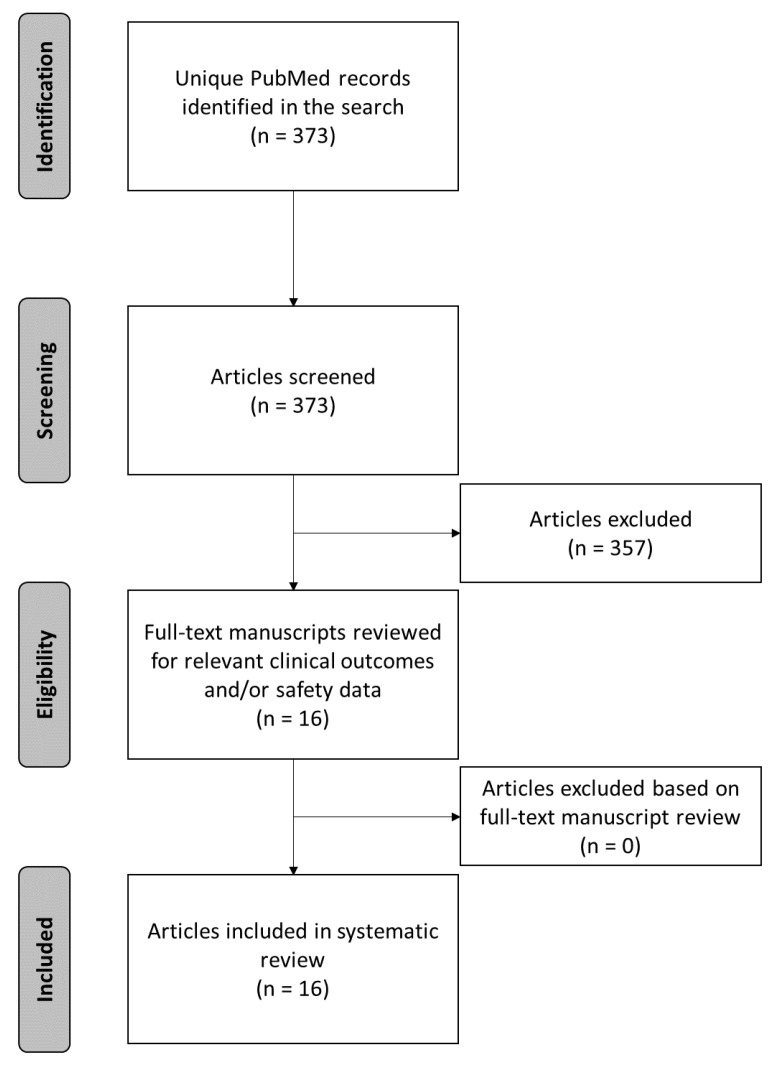
Study flow diagram.

A total of 373 unique articles were identified from the initial PubMed query: 327 articles were screened by title and/or abstract only, and 46 articles were screened by full-text manuscript review (see Figure 1). In total, 357 articles were excluded. The full-texts of the 16 remaining articles were subsequently reviewed for the presence of relevant effectiveness and/or safety outcomes. All 16 articles were deemed suitable for inclusion in the systematic review. Studies were organized by the predominant lead location (thoracic, cervical, or combined thoracic and cervical) and follow-up duration (≤12 months and >12 months), and are described in narrative form below.

### 3.2. Study Characteristics

Table 1 provides an overview of key characteristics for the 16 selected studies, of which 11 were single-center studies, five were multicenter studies, and 14 were judged to include consecutive patients based upon statements relating to inclusion or whether the cohort appeared to encompass all patients of interest. The articles were published between 2015 and 2020. Eight studies were conducted in the USA, and two each in Australia, Germany, and the UK. The remaining two studies were multinational (Germany, UK, and the USA; and Belgium, Germany, and The Netherlands). Among the 16 studies, 15 reported effectiveness outcomes, and 11 presented safety outcomes. Follow-up duration ranged from 6 months to over 2 years. Effectiveness and safety outcomes are summarized in Table 2 and Table 3, respectively.

### 3.3. Effectiveness Outcomes with Predominant Thoracic Lead Placement

In 12 studies that reported effectiveness outcomes, the majority of patients had 10 kHz SCS leads placed in the thoracic region, usually to treat pain distributed in their back and/or leg(s). Stauss et al. (2019) conducted the largest study, including 1660 trunk and/or limb pain patients from eight centers in three countries [55], while Sills (2020) evaluated effectiveness outcomes for the longest follow-up duration (30 months) in their single-center study [49].

#### 3.3.1. Studies with ≤12 Months of Follow-Up

In Stauss and colleagues’ study, 84% of trialed and/or implanted patients (1370/1640) had chronic back and leg pain [55]. Final follow-up data were available for 1131 patients at a mean of 8.9 ± 6.7 months (range: 0.1–33.2). The authors reported a reduction in average pain intensity on a verbal numerical rating scale (VNRS) of 63% from baseline among the cohort, and a responder rate of 74% (838/1131) based on patient-reported percentage pain relief during their last visit follow-up (up to 33.2 months). During the follow-up period, 32% of patients (343/1070) decreased their medication intake. A general improvement in function and sleep was noted in 72% (787/1088) and 68% (694/1020) of patients, respectively. Outcomes were also analyzed in a subgroup of 266 patients with previously failed traditional LF-SCS. At a mean of 10.7 ± 7.7 months, the analysis found the same average reduction in pain intensity (63%) and responder rate as the full cohort (74%; 197/266). Similarly, 33% of this group (13/40) decreased their medication intake, 83% reported a general improvement in function (33/40), and 70% noted improved sleep (21/30).

In a smaller, multicenter, Australian study by Russo et al. (2016), the majority of included patients (N = 256) also had back and/or leg pain (69%; 177/256), with leads typically placed from vertebral levels T8 to T11 [59]. In total, 186 patients (73%) received permanent systems. After 6 months of treatment, pain intensity decreased by an average of 51% on a numerical rating scale (NRS; *p* < 0.001; N = 125). Disability among those with available data was also noted to improve by 8.6 points on the Oswestry Disability Index (ODI; *p* < 0.001; N = 68). In a subgroup of 38 patients who had previously undergone failed traditional LF-SCS and/or peripheral nerve field stimulation (PNFS), mean pain reduction was similar to the full cohort at the 6-month assessment (49% vs. 51%, respectively; both *p* < 0.001). The authors also reported ≥50% pain relief in 55% of this subgroup.

In another Australian study, Finch et al. (2018) performed an audit of 58 patients treated in their clinic with 10 kHz SCS up to 12 months after implantation [51]. The majority (84%; 49/58) were treated for spinal pain, with leads spanning T9-T10. Average pain intensity in the group decreased significantly on a visual analog scale (VAS) between baseline and 12 months (*p* < 0.001), and disability score improved on the ODI by 13.8 points (*p* < 0.001; N = 56).

Another single-center study conducted in a community-based pain facility by DiBenedetto et al. (2018) evaluated 12-month outcomes in 32 patients treated with 10 kHz SCS for back pain with or without leg pain [56]. The investigators found an average decrease in back pain on the NRS of 46% (*p* < 0.001; N = 30) and a similar mean reduction in leg pain (51%; *p* = 0.010; N = 16). In total, 71% of patients (15/21) decreased their opioid dosage during the follow-up period, with an average reduction of 28% (*p* = 0.001; N = 21). Disability score also improved by an average of 3.1 points over the 12 months among those who completed the modified Roland Morris Disability Questionnaire (RMDQ; *p* = 0.020; N = 21).

The efficacy of 10 kHz SCS in patients with thoracic back pain was analyzed in a multicenter study by Sayed et al. (2020) [47]. All 19 implanted patients had at least one lead placed between vertebral levels T1 and T6. At the 12-month follow-up, nine patients with available data experienced an average decrease in NRS pain score of 70% (*p* = 0.004). At their last follow-up, response to therapy was observed in 89% of patients (17/19), and 47% (9/19) decreased their medication intake. In total, 84% (16/19) and 74% (14/19) of the cohort also indicated general improvements in function and sleep, respectively.

Gill et al. (2019) evaluated 10 kHz SCS outcomes in 12 implanted patients from their clinic with complex regional pain syndrome (CRPS) [52]. Patients had lower (n = 10) or upper (n = 2) extremity symptoms, with leads placed accordingly at vertebral levels T8–12 or C2-C7. At a mean follow-up time of 12.1 ± 4.6 months, two thirds of the group (8/12) were classified as responders based upon patient-reported percentage pain relief. Among the subset of seven patients with previously failed traditional LF-SCS (due to inadequate response or paresthesia side effects), 71% (5/7) responded to 10 kHz SCS, with an average patient-reported percentage pain relief of 58%.

Schieferdecker et al. (2019) noticed that 10 kHz SCS therapy positively affected bladder incontinence in some of their patients. The authors reviewed data from five trunk and/or limb pain patients in their clinic treated with 10 kHz SCS who also had neurogenic bladder dysfunction secondary to either spinal injury during surgery (n = 4) or demyelinating neurological disease (n = 1) [54]. Activated leads covered the T8-T9 or T9-T10 vertebral levels. Some patients also had cervical leads implanted; however, stimulation leads in this region were inactive. Patients were followed for an average of 10 months (range: 5–14). Among the four patients who reached 6 months of follow-up at the time of reporting, the average reduction in pain intensity on an NRS was 56%, and all experienced ≥50% pain relief (i.e., responded). Similarly, the two patients who reached 12 months of follow-up were both responders, with average pain relief of 53%. Furthermore, all five patients reported substantially decreased incontinence frequency (mean: 80%), as well as an average improvement in QoL score of 38% on the ICIQ-LUTSqol instrument (International Consultation on Incontinence Questionnaire Lower Urinary Tract Symptoms Quality of Life).

High-frequency 10 kHz SCS has also been reported by Simopoulos et al. (2018) to improve neuropathic pelvic pain in a small case series from their clinic comprising three patients [57]. Individual diagnoses included coccydynia, post-laminectomy cauda equina syndrome, and pudendal neuralgia. Leads were placed at the superior endplate of the T8 vertebral level and mid-T9 vertebral level. At a mean follow-up of 10.7 months (range: 9–12), the group reported an average reduction in pain of 53% on the VAS, and two of the three patients continued to respond to therapy. One of the responders was able to reduce their opioid intake by 75%.

#### 3.3.2. Studies with >12 Months of Follow-Up

Al-Kaisy et al. (2020) exclusively focused on patients with back pain with or without leg pain in their single-center study that examined the effects of 10 kHz SCS delivered via sequentially activated adjacent bipoles (cascade programming) covering vertebral levels T8-T10 in 99 implanted patients [45]. In total, 72 patients surpassed 12 months of follow-up (average: 15.1 ± 4.2 months) and reported average NRS reductions in back pain and leg pain of 52% (*p* < 0.0001; N = 72) and 53% (*p* < 0.0001; N = 58), respectively. Corresponding responder rates were 56% (40/72) and 59% (34/58). The majority of the cohort (83%; 60/72) also indicated they were moderately to a great deal better on the Patient Global Impression of Change (PGIC) questionnaire.

While three of the studies described above analyzed 10 kHz SCS outcomes in subgroups of patients with previously failed traditional LF-SCS [52,55,59], Ghosh et al. (2020) focused solely on this challenging cohort [46]. Patients from their center with previously failed traditional LF-SCS were principally diagnosed with either failed back surgery syndrome (FBSS; 61%; 17/28) or CRPS (32%; 9/28). The median follow-up time was 21.2 ± 8.4 months. Among the patients with a clinically important reduction in NRS score, the average reduction in pain intensity on the NRS was 64% (*p* < 0.0001; N = 13), with FBSS and CRPS patients experiencing 60% (*p* < 0.0001; N = 6) and 52% (*p* < 0.0001; N = 6) mean pain relief, respectively. Corresponding responder rates for all, FBSS, and CRPS patients were 46% (13/28), 35% (6/17), and 67% (6/9), respectively. Among FBSS patients, average disability score on the ODI decreased by 19.7 points (*p* < 0.0001; N = 17) during the follow-up period, and 44% reduced their ODI disability category from severe and/or bedbound to minimal and/or moderate.

Sills (2020) reported the outcomes of lower body polyneuropathic pain treated with 10 kHz SCS at his center [49]. Clinical outcomes were assessed at a mean of 29.8 months (range: 25–38) in six patients. Diagnoses included painful diabetic polyneuropathy (PDPN; n = 3), idiopathic polyneuropathy (iPN; n = 2), and chronic inflammatory demyelinating polyneuropathy (CIDP; n = 1). Stimulation was targeted near vertebral levels T9-T10. At their last follow-up, the cohort reported an average reduction in VNRS pain intensity of 60%, and half had a decrease in pain of at least 50%. Notably, among the three PDPN patients, two experienced pain relief of >90% (versus 33% in iPN patients), and all three reported improved sensation relative to baseline. Furthermore, two-thirds of the cohort (4/6) had eliminated or reduced their opioid consumption by their last follow-up.

### 3.4. Effectiveness Outcomes in Studies with Predominant Cervical Lead Placement

Three studies reported effectiveness outcomes in patient populations in which the majority had 10 kHz SCS leads placed in the cervical region. Of these, two studies evaluated effectiveness in neck and/or upper limb pain patients [48,50]. The remaining study focused on patients with neuropathic limb pain, most of whom had upper limb pain symptoms (73%; 8/11) [60].

#### 3.4.1. Studies with ≤12 Months of Follow-Up

In the first neck and/or upper limb pain study by El Majdoub et al. (2019), 24 patients from their clinic were trialed with 10 kHz SCS via a single cervical lead with the tip located at the C2 vertebral level [50]. Of the 23 permanently implanted patients, 20 reached the 12-month follow-up at the time of analysis. The group reported, on average, a 74% (*p* < 0.01) and 77% (*p* < 0.05) decline in neck pain and upper limb pain on the VAS, respectively. Average functional capacity measured on the ODI and Global Assessment of Function (GAF) scales also improved, with a mean decrease of 11.2 points and median interval increase from 50–41% to 70–61%, respectively. Among the 23 patients who took opioids at baseline, total daily opioid dosage decreased from 1020 mg/day to 450 mg/day at 12 months, a reduction of 56%. Among the 10 patients who took nonsteroidal anti-inflammatory drugs (NSAIDs) at baseline, the total daily dosage decreased from 6750 mg/day to 1425 mg/day at 12 months, a reduction of 79%.

Al-Kaisy et al. (2015) investigated 10 kHz SCS for neuropathic limb pain in a single-center evaluation [60]. The majority of the 11 implanted patients (73%; 8/11) had upper limb pain, with leads placed at the C2-C7 vertebral levels. The remainder (27%; 3/11) had lower limb pain, with leads positioned at the T8–12 vertebral levels. After 6 months of treatment, average NRS pain intensity declined by 59% (*p* < 0.050), and 73% (8/11) achieved response to therapy. During the follow-up period, the authors also noted an average improvement of 101% in QoL score among the cohort, measured using the EuroQol-5D (EQ-5D) time trade-off valuation, as well as a 79% reduction in catastrophic thinking related to pain (Pain Catastrophizing Scale [PCS]). The average intensity and impact of pain also improved by 49% (N = 10) on the Brief Pain Inventory (BPI).

#### 3.4.2. Studies with >12 Months of Follow-Up

In the second of the studies examining outcomes in neck and/or upper limb pain, Sayed et al. (2020) reviewed multicenter data from 47 patients at a median follow-up of 19.4 ± 12.4 months [48]. Leads were placed at locations ranging from vertebral levels C2 to C6. At the last follow-up, average patient-reported percentage pain relief was 58% (N = 46), and 76% (35/46) achieved response to therapy. The authors further examined pain relief and responder status according to whether patients were surgery naïve or not, and found similar outcomes regardless of surgical history (mean patient-reported pain relief: 59% [N = 24] vs. 60% [N = 18], respectively; responder rate: 71% [17/24] vs. 83% [15/18], respectively). Among the whole cohort, 36% (17/47) decreased their medication intake, 72% (34/47) reported improved function, and 53% (25/47) indicated improved sleep.

### 3.5. Effectiveness Outcomes in Studies with Combined Thoracic and Cervical Lead Placement

Combined cervical and thoracic lead placement (C2 + T2, C2 + T9, or C2 + T2 + T9) was evaluated in only one study. Salmon (2019) evaluated 10 kHz SCS in a population of 38 patients from his clinic with widespread neuropathic/nociplastic pain, followed for a mean of 2.3 ± 1.7 years [53]. Among the 35 patients using their device at the last follow-up, average overall NRS pain intensity decreased by 48% (*p* = 0.00001). The author also noted 63%, 60%, and 59% reductions in head and neck pain, upper back pain, and lower back pain, respectively. Furthermore, opioid consumption decreased by 40% within the group of 15 patients who remained on opioids at the last follow-up (down from 24 patients at baseline). Among those on high dose opiates (N = 11), opioid intake declined by 47%. Among the main study population, functional improvement was observed on the RMDQ (mean reduction: 4.5 points; *p* ≤ 0.050; N = 29). Both the pain self-efficacy (PSEQ) and anxiety and depression (DASS) instruments also indicated psychometric benefits, with an average increase in PSEQ score of 13 points (N = 29), and fewer patients categorized as moderately severe or severe on the DASS compared with baseline (moderately severe: 3.6% vs. 6.9%; severe: 7.1% vs. 10.3%). The majority (79%; 23/29) of patients indicated they were moderately to a great deal better on the PGIC questionnaire. Notably, the proportion of work-eligible patients in employment more than doubled compared with baseline (20/31 vs. 8/31).

### 3.6. Safety Outcomes

#### 3.6.1. Lead Migration

Safety data were reported in 11 studies and are summarized in Table 3. Of these, five studies reported details of lead migration. The incidence of lead migration in these studies in 10 kHz SCS patients ranged from 0% to 7.1% for leads predominantly placed in the thoracic region [45,52,59], and from 4.3% to 18.2% for leads predominantly located in the cervical area [50,60]. In total, 13 of 331 patients were affected, and all but 1 of 10 with available details underwent revision surgery.

#### 3.6.2. Infection

The incidence of infection, including during the trial period, ranged from 0% to 13% in six studies [45,46,50,52,59,60]. In total, 9 of 378 patients experienced an infection. Of these, five developed infections during the trial period and had their systems explanted, with permanent systems implanted or planned later; three had their systems explanted; and one had no details available.

#### 3.6.3. Pain over the Site of the Implantable Pulse Generator

Pain over the site of the implantable pulse generator (IPG) occurred at a rate of 0% to 27.3% in six studies [45,48,52,53,59,60]. In total, 28 of 393 patients were affected, 17 of whom were managed conservatively or required no intervention, nine of whom required surgical intervention, and two had no details available.

#### 3.6.4. Insufficient Pain Relief/Nonresponders/Treatment Failure

The incidence of insufficient pain relief ranged from 0% to 15.8% in four studies [48,52,53,59]. In total, 9 of 283 patients were affected, two of whom underwent reprogramming to resolve their issue, six of whom had additional epidural leads placed to improve pain relief, and one had no details available.

#### 3.6.5. Lead Fracture

Lead fracture or high impedance incidence rates were 0% and 2.6% in two studies that examined safety outcomes in a total of 50 patients [52,53]. The single affected patient underwent lead replacement.

#### 3.6.6. Neurological Injury

Importantly, neurological deficit was not reported by any study, while one study documented increased sensation in several patients with painful polyneuropathy, indicating neurological improvement [49].

#### 3.6.7. System Explantation

In the first of two multinational studies that detailed 10 kHz SCS explant rates, Stauss et al. (2019) reported 48 explants among 1290 patients, an incidence rate of 3.7% [55]. Of these, 22 systems were explanted due to infection, 15 due to loss of efficacy, and 11 for other reasons. Corresponding incidence rates were 1.7% (22/1290), 1.2% (15/1290), and 0.9% (11/1290), respectively. In the second study, Van Buyten et al. (2017) analyzed therapy-related explants among 955 SCS systems implanted over 3 years [58]. Of these, 155 implants were 10 kHz SCS systems, with a mean follow-up of 2.83 years per implant. Of these, 22 systems were explanted for insufficient pain relief, an annual incidence of 5.0% (95% CI, 3.3–7.6%).

## 4. Discussion

Several literature reviews of 10 kHz SCS therapy have summarized treatment effectiveness. However, these reviews were not conducted systematically and did not examine safety outcomes in detail [41,42,43,44]. This review is the first to systematically examine both the efficacy and safety of 10 kHz SCS under real-world conditions.

The evidence identified during our literature search included 16 studies in total, encompassing 2382 patients implanted with a 10 kHz SCS system. Follow-up ranged from 6–34 months. The patient populations mainly had pain located in their back and/or legs, neck and/or upper limbs, or purely in their upper or lower extremities. Leads were generally placed at the T8-T12 vertebral levels to treat pain in the back and/or legs, while pain in the neck and/or upper limbs was usually treated via C2-C7 lead placement. All but one of the 15 studies that included effectiveness outcomes reported either average pain relief or responder rate (or at least one of these values could be derived from the data presented). Studies that reported safety outcomes (11/16) identified complications associated with the therapy, sequelae, and the number of patients affected. Nine studies provided information on medication use, nine detailed changes in functional capacity, and eight presented QoL data. Notably, more than two-thirds of the studies were published within the last 2 years, and a third within the last 6–7 months, showing the rapid emergence of new evidence in this field.

### 4.1. Effectiveness Outcomes

At ≤12 months of follow-up, average pain relief across the studies (not including subgroups) ranged from 46–77% [47,50,54,55,56,57,59,60], and responder rates from 67–100% [47,52,54,55,57,60]. Beyond 12 months of follow-up, mean pain relief and responder rate ranged from 48–64% [45,46,48,49,53] and 46–76%, respectively [45,46,48,49]. In six studies, 32–71% of patients decreased their opioid or medication intake at 9–30 months of follow-up [47,48,49,53,55,56]. Improved functional capacity on the ODI or RMDQ instruments was also observed in six studies [46,50,51,53,56,59], with 72–84% of patients in three additional studies reporting general improvements in function [47,48,55]. Furthermore, three studies documented QoL gains across various scales including incontinence frequency, ICIQ-LUTSqol, EQ-5D time trade-off valuation, PCS, BPI, PSEQ, DASS, and work participation [53,54,60]. Seven studies also reported general QoL improvements including in sleep, sensation, and PGIC rating [45,47,48,49,53,54,55].

Overall, our synthesis of the retrospective evidence on 10 kHz SCS found that pain relief was sustained at long-term follow-up, and a significant proportion of patients were able to reduce their opioid or medication requirements. The combination of durable pain relief and opioid reduction associated with 10 kHz SCS is an important consideration given the questionable benefit and risks attendant with long-term opioid use in chronic pain patients. In addition, most studies in our review also reported improved functional capacity and/or QoL. This has the potential to reduce health care costs among this often highly disabled group of patients. Moreover, studies in our review that examined subgroups of patients with a history of failed traditional LF-SCS found similar pain relief outcomes to patients who were SCS-naïve, suggesting that 10 kHz SCS may be a useful salvage therapy in this difficult-to-treat cohort. Ultimately an SCS national/global registry with 100% compliance would more accurately capture the long-term safety and efficacy of these devices.

### 4.2. Safety Summary

The most common complication associated with 10 kHz SCS among the studies that provided safety data was IPG site pain with a mean incidence across studies of 10.4%, followed by lead migration (6.2%), insufficient pain relief (5.1%), infection (4.0%), and lead fracture (1.3%). The incidence rates of these complications are consistent with the published SCS literature (Table 4) [61,62,63,64,65,66,67]. Only one study reported an overall explant rate (3.7%) [55], which was lower than rates recently published for SCS (18.8% to 23.9%) [58,68]. Indeed, true real-world explant rates are urgently needed to provide a more accurate account of the safety profile of this technology. This should be encouraged in future research and would help with reducing the likelihood of underreported explantation rates. Importantly, no permanent neurological deficits associated with 10 kHz SCS were reported by any of the included studies.

### 4.3. Strengths and Limitations

This review provides a systematic and comprehensive overview of the effectiveness and safety data from all 10 kHz SCS retrospective studies. The included research was conducted in multiple countries and institutions, and included pragmatic chronic pain populations. Follow-up duration in several studies exceeded that of the longest duration RCT and formal prospective studies of 10 kHz SCS in selected populations carried out to date. Finally, since more than half of the studies were published within the last 2 years, and a third were published within the last 6–7 months, this review captures the most recent therapy outcomes in a rapidly evolving area of evidence.

There are some limitations to this review. The included studies were retrospective in design and were therefore limited by potential selection biases as well as the availability and accuracy of recorded data. In addition, most studies were conducted in one or two centers, follow-up was not uniform, outcomes were not reported longitudinally, remission data were absent, and sample sizes were small in around half of the studies. Furthermore, the reporting of medication, functional capacity, QoL, and safety data were inconsistent across studies and not at a granular level. Meaningful categorization of the studies in terms of the chronic pain diagnosis was also challenging due to the heterogeneity of the patient populations, for example, the wide range of pain distribution (cervical, thoracic, and lumbar). Therefore, a meta-analysis was not considered appropriate. The limitations of retrospective studies generally should be considered during interpretation of the results.

## 5. Conclusions

The application of high-frequency spinal cord stimulation in the management of chronic pain is rapidly evolving, with multiple studies published very recently evaluating the 10 kHz SCS modality. This systematic review and narrative synthesis provides a comprehensive and up-to-date overview of all retrospective real-world studies to aid clinical decision making in routine practice. Our results suggest that 10 kHz SCS provides durable pain relief and can facilitate a reduction in the use of pain-relieving medication (including opioids). Furthermore, the therapy appears to be associated with improved functional capacity and quality of life. 10 kHz SCS has a safety profile comparable to current and historical devices.

## Figures and Tables

**Table 1:** Study characteristics.

Reference	Year Published	Single/ Multicenter	Consecutive Patients	Follow-Up Duration	Key Inclusion	Outcomes Reported	Geographical Location
Al-Kaisy et al. [45]	2020	Single center	Yes	15.1 ± 4.2 mo	Back pain with or without leg pain and programmed using Cascade protocol	NRS, responder rate, trial-to-perm ratio, and PGIC.	UK
Ghosh et al. [46]	2020	Single center	Yes	21.2 ± 8.4 mo	Failed traditional SCS trial or permanent implant	NRS, responder rate, trial-to-perm ratio, ODI, and SFMPQ.	USA
Sayed et al. [47]	2020	Multicenter	Yes	12 mo	Thoracic back pain and lead(s) placed between T1–T6	NRS, responder rate, general change in function and sleep, and change in medication.	USA
Sayed et al. [48]	2020	Multicenter	Yes	19.4 ± 12.4 mo	Neck and/or upper limb pain	VRS, patient-reported percentage pain relief, responder rate, general change in function and sleep, and change in medication.	USA
Sills [49]	2020	Single center	Yes	29.8 mo	Peripheral neuropathy	VNRS, responder rate, trial-to-perm ratio, change in sensation, change in medication, and general improvement.	USA
El Majdoub et al. [50]	2019	Single center	Yes	12 mo	Neck and/or upper limb pain	VAS, trial-to-perm ratio, ODI, and GAF, change in medication, and satisfaction.	Germany
Finch et al. [51]	2019	Single center	Yes	12 mo	Chronic pain and implanted with a 10 kHz SCS system	VAS, ODI, and change in medication.	Australia
Gill et al. [52]	2019	Single center	Yes	12.1 ± 4.6 mo	Uni- or bilateral CRPS	NRS, patient-reported percentage pain relief, responder rate, trial-to-perm ratio, and SF-MPQ-2.	USA
Salmon [53]	2019	Single center	Yes	2.3 ± 1.7 y	Combined upper and lower body neuropathic/nociplastic pain syndromes	NRS, trial-to-perm ratio, RMDQ, PGIC, PSEQ, DASS, satisfaction, opioid use, and employment capacity.	USA
Schieferdecker et al. [54]	2019	Single center	No	10.0 mo	Trunk and/or limb pain + neurogenic bladder dysfunction	NRS, ICIQ-LUTSqol, micturition frequency, incontinence frequency, residual volume, and catheterization frequency.	Germany
Stauss et al. [55]	2019	Multicenter	Yes	8.9 ± 6.7 mo	Trunk and/or limb pain	VNRS, patient-reported percentage pain relief, responder rate, trial-to-perm ratio, changes in medication use, general change in function and sleep, general QoL, and satisfaction.	Germany, UK, and USA.
DiBenedetto et al. [56]	2018	Single center	Yes	12 mo	Back pain with or without leg pain	Daily MME, visit volume, functional pain scale, NRS, PCS, PHQ-9, PHQ-15, Generalized Anxiety Disorder-7, WHODAS 2.0, and RMDQ-m.	USA
Simopoulos et al. [57]	2018	Single center	No	10.7 mo	Neuropathic pelvic pain	VAS	USA
Van Buyten et al. [58]	2017	Multicenter	Yes	2.83 y	Implanted with SCS device for dorsal column stimulation	Explants	Belgium, Germany, and the Netherlands.
Russo et al. [59]	2016	Multicenter	Yes	6 mo	Not candidates for SCS or nonresponders	NPRS, responder rate, trial-to-perm ratio, ODI, and activity tolerance.	Australia
Al-Kaisy et al. [60]	2015	Single center	Yes	6 mo	Neuropathic pain in the upper or lower limbs	NRS, responder rate, trial-to-perm ratio, BPI, PCS, EQ-5D, painDETECT, and satisfaction.	UK

BPI: Brief Pain Inventory; CRPS: Complex Regional Pain Syndrome; DASS: Depression, Anxiety and Stress Scale; EQ: EuroQol; GAF: Global Assessment of Function; ICIQ-LUTSqol: International Consultation on Incontinence Questionnaire Lower Urinary Tract Symptoms Quality of Life; MME: Morphine Milligram Equivalent; NRS: Numerical Rating Scale; mo: months; ODI: Oswestry Disability Index; PCS: Pain Catastrophizing Scale; PGIC: Patient Global impression of Change; PHQ: Patient Health Questionnaire; PSEQ: Pain Self Efficacy Questionnaire; QoL: Quality of life; RMDQ: Roland Morris Disability Questionnaire; SFMPQ: Short-form McGill Pain Questionnaire; VAS: Visual Analog Scale; VNRS: Verbal Numerical Rating Scale; VRS: Verbal Rating Scale; WHO-DAS: World Health Organization-Disability Assessment Schedule; y: years.

**Table 2:** Study effectiveness data.

Reference	Year	N Implanted Subjects	Pain Location	Lead Location	Responder Rate % (n/N)	Average Pain Relief % (N)	Opioid/Medication Change from Baseline to Last Follow-Up	Patients that Reduced or Eliminated Opioids/Medication at Last Follow-Up (%, n/N)	Average Functional Change from Baseline to Last Follow-Up (N)	Patients that Improved Functional Outcomes at last Follow-Up (%, n/N)	Average QoL Change from Baseline to Last Follow-Up (N)	Patients that Improved QoL Outcomes at Last Follow-Up (%, n/N)
Stauss et al. [55]	2019	1660	Back and leg pain: 84% (1370/1640) Other: 16% (270/1640)	NR (Back and/or leg pain: Typically T8-T12)	All patients: 74% (838/1131) Previous LF-SCS subgroup: 74% (197/266)	All patients: 63% (NR) Previous LF-SCS subgroup: 63% (N = 266)	NR	All patients 32% (343/1070) Previous LF-SCS subgroup: 33% (13/40)	NR	General improvement in function: All patients: 72% (787/1088) Previous LF-SCS subgroup: 83% (33/40)	NR	General improvement in sleep: All patients: 68% (694/1020) Previous LF-SCS subgroup: 70% (21/30)
Russo et al. [59]	2016	186	Back and/or leg: 69% (177/256) Head ± neck: 8% (21/256) Neck ± arm/shoulder: 6% (15/256) Other/ unrecorded: 17% (43/256)	Low back and/or leg pain: T8-T11 Neck and arm pain: C2/3 disc, C3, C4	All patients: NR Previous SCS/PNFS subgroup: 55% (21/38)	All patients: 51% (*p* < 0.001; N = 125) Previous SCS/PNFS subgroup: 49% (*p* < 0.001; N = 38)	NR	NR	ODI score: All patients: 41.4 to 32.8 points (21% reduction; *p* < 0.001; N = 68) Previous SCS/PNFS subgroup: NR	NR	NR	NR
Finch et al. [51]	2019	58	Spinal: 84% (49/58) Other: 16% (9/58)	T9-T10: 93% (54/58) T9-T10 + T1-T2: 3% (2/58) T9-T10 + C2: 3% (2/58)	NR	NR (*p* < 0.001; N = 58)	Change from baseline to 3–6 months clinical review: 72.7 to 62.8 mg/day MEDD (*p* < 0.05, N = 57)	NR	Change from baseline to 3–6 months clinical review in ODI score: 50.4 to 36.6 points (27% reduction; *p* < 0.001; N = 56)	NR	NR	NR
DiBenedetto et al. [56]	2018	32	Back ± leg	NR (Back and/or leg pain: Typically T8-T12)	Back pain: NR Leg pain: NR	Back: 46% (*p* < 0.001; N = 30) Leg: 51% (*p* = 0.01; N = 16)	92.2 to 66.0 mg/day MEDD (28% reduction; *p* = 0.001; N = 21)	71% (15/21)	RMDQ-m score: 13.9 to 10.8 points (22% reduction; *p* = 0.02; N = 21) WHO-DAS score: 1.97 to 1.92 points (*p* = 0.57; N = 19)	NR	NR	NR
Sayed et al. [47]	2020	19	Thoracic back	T1-T6	89% (17/19) (Last-follow-up)	70% (*p* = 0.004; N = 9)	NR	47% (9/19)	NR	General improvement in function: 84% (16/19)	NR	General improvement in sleep: 74% (14/19)
Gill et al. [52]	2019	12	Lower extremities: 83% (10/12) Upper extremities: 17% (2/12)	T8–T12: 83% (10/12) C2–C7: 17% (2/12)	All patients: 67% (8/12) Previous LF-SCS subgroup: 71% (5/7)	All patients: NR Previous LF-SCS subgroup: 58% (N = 7)	NR	NR	NR	NR	NR	NR
Schieferdecker et al. [54]	2019	5	Back and/or legs: 80% (4/5) Other: 20% (1/5)	T9-T10 (4/5) T8-T9 (1/5)	6 mo: 100% (4/4) 12 mo: 100% (2/2)	6 mo: 56% (N = 4) 12 mo: 53% (N = 2)	NR	NR	NR	NR	Incontinence frequency: 80% reduction (N = 5) ICIQ-LUTSqol score: 38% reduction (N = 5)	Incontinence frequency: 100% (5/5) ICIQ-LUTSqol score: 100% (N = 5)
Simopoulos et al. [57]	2018	3	Pelvis	T8 superior endplate + mid-T9	67% (2/3)	53% (N = 3)	n = 1 reported a 75% reduction in opioids	NR	NR	NR	NR	NR
Al-Kaisy et al. [45]	2020	99	Back ± leg	T8-T10	Back pain: 56% (40/72) Leg pain: 59% (34/58)	Back: 52% (*p* < 0.0001; N = 72) Leg: 53% (*p* < 0.0001; N = 58)	NR	NR	NR	NR	NR	PGIC scale: 83% of patients moderately to a great deal better (60/72)
Ghosh et al. [46]	2020	28	FBSS: 61% (17/28) CRPS: 32% (9/28) Other (neck, groin, or rectal pain): 7% (2/28)	NR (Back and/or leg pain: Typically T8-T12)	All patients: 46% (13/28) FBSS subgroup: 35% (6/17) CRPS subgroup: 67% (6/9)	All responding patients *: 64% (*p* < 0.0001; N = 13) FBSS subgroup *: 60% (*p* < 0.0001; N = 6) CRPS subgroup *: 52% (*p* < 0.0001; N = 6)	NR	NR	ODI score: FBSS subgroup: 58.3 to 38.6 points (34% reduction; *p* < 0.0001; N = 17)	ODI category: FBSS subgroup: 44% reduced their disability category from severe and/or bedbound to minimal and/or moderate	NR	NR
Sills [49]	2020	6	Lower extremities ± feet: 67% (4/6) Legs + feet: 17% (1/6) Low back + legs: 17% (1/6)	Stimulation near T9-T10	50% (3/6)	60% (N = 6)	NR	67% (4/6)	NR	NR	NR	Sensation: 67% (4/6) reported improvements (3 PDPN; 1 iPN)
Al-Kaisy et al. [60]	2015	11	Upper limb: 73% (8/11) Lower limb: 27% (3/11)	C2–C7: 73% (8/11) T8–T12: 27% (3/11)	73% (8/11)	59% (*p* < 0.05; N = 11)	NR	NR	NR	NR	EQ5D time trade off score: 101% increase (N = 11) PCS score: 33 to 7 points (79% reduction; N = 11) BPI score: 57.6 to 29.4 points (49% reduction; N = 10)	NR
El Majdoub et al. [50]	2019	23	Neck and/or upper limb	C2	NR	Neck pain: 74% (*p* < 0.01; N = 20) Upper limb: 77% (*p* < 0.05; N = 20)	Opioids: 1020 to 450 mg/day morphine equivalent total daily dosage (56% reduction; N = 23) NSAIDs: 6750 mg/day to 1425 mg/day total daily dosage (79% reduction; N = 10)	NR	ODI score: 31.0 to 19.8 points (36% reduction, N = 20) GAF median interval: 50–41% to 70–61% (N = 20)	NR	NR	NR
Sayed et al. [48]	2020	47	Neck and/or upper limb	C2-C6	All patients: 76% (35/46) Surgery naïve: 71% (17/24) Previous spine surgery: 83% (15/18)	All patients: 58% (N = 46) Surgery naïve: 59% (NR) Previous spine surgery: 60% (NR)	NR	All patients: 36% (17/47)	NR	General improvement in function: All patients: 72% (34/47)	NR	General improvement in sleep: 53% (25/47)
Salmon [53]	2019	38	Truncal/spinal regions + distal extremities	C2 + T9: 37% (13/35) C2 + T2 + T9: 37% (13/35) C2 + T2: 26% (9/35)	NR	All patients: 48% (*p* = 0.00001; N = 35) Head and neck pain subgroup: 63% (NR) Upper back pain subgroup: 60% (NR) Lower back pain subgroup: 59% (NR)	Patients on opioids at last follow-up: 165.4 to 99.3 mg/day MEDD (40% reduction; N = 15) Patients on high-dose opioids: 210.5 to 111.8 mg/day MEDD (47% reduction; N = 11)	38% (9/24)	RMDQ score: 12.3 to 7.8 points (37% reduction; *p* ≤ 0.05; N = 29)	NR	PSEQ score:21.0 to 34.0 points (62% increase; N = 29) DASS: 6.9% to 3.6% of patients classified as moderately severe (N = 29) 10.3% to 7.1% of patients classified as severe (N = 29) Work participation in work eligible patients: 8/31 to 20/31 patients	PGIC: 79% moderately to a great deal better (23/29)

*, study reported NRS scores in patients who achieved clinically important reduction in pain intensity. BPI: Brief Pain Inventory; CRPS: Complex Regional Pain Syndrome; DASS: Depression, Anxiety and Stress Scale; EQ: EuroQol; FBSS: Failed Back Surgery Syndrome; GAF: Global Assessment of Function; ICIQ-LUTSqol: International Consultation on Incontinence Questionnaire Lower Urinary Tract Symptoms Quality of Life; iPN: Idiopathic Polyneuropathy; LF-SCS: Low-Frequency Spinal Cord Stimulation; MEDD: Morphine Equivalent Daily Dose; NR: Not Reported; NRS: Numerical Rating Scale; NSAID: Nonsteroidal Anti-Inflammatory Drug; ODI: Oswestry Disability Index; PCS: Pain Catastrophizing Scale; PDPN: Painful Diabetic Polyneuropathy; PGIC: Patient Global impression of Change; PNFS: Peripheral Nerve Field Stimulation; PSEQ: Pain Self Efficacy Questionnaire; RMDQ: Roland Morris Disability Questionnaire; SCS: Spinal Cord Stimulation; WHO-DAS: World Health Organization - Disability Assessment Schedule.

**Table 3:** Study safety data.

Reference	Year Published	Safety Population N	Adverse Event	Affected Patients n (%)	Status at Time of Reporting
Stauss et al. [55]	2019	1290	Explant due to infection	22 (1.7%)	-
			Explant due to loss of efficacy	15 (1.2%)	-
			Explant for other reasons	11 (0.9%)	-
Russo et al. [59]	2016	186	Lead migration	3 (1.6%)	NR
			IPG /anchor site pain	2 (1.1%)	NR
			Infection	1 (0.5%)	NR
			Loss of therapy efficacy	1 (0.5%)	NR
Van Buyten et al. [58]	2017	155	Explant due to ineffective stimulation	22 (14.2%) *	-
Al-Kaisy et al. [45]	2020	114	Infection during trial	4 (3.5%)	4/4 systems explanted with permanent implantations carried out later
		99	IPG site pain	16 (16.2%)	9/16 had surgical intervention; 7/16 managed conservatively
			Lead migration	7 (7.1%)	6/7 had leads revised; 1/7 elected not to have revision
			Suspected hardware malfunction	1 (1.0%)	1/1 system replaced
			Infection after permanent implantation	0 (0.0%)	-
Finch et al. [51]	2019	58	All complications, including wound infection, hematoma, lead migration, and IPG repositioning	21 (36.2%)	NR
Sayed et al. [48]	2020	47	Insufficient pain relief	2 (4.3%)	2/2 resolved with programming
			Overstimulation	2 (4.3%)	2/2 resolved with programming
			IPG site pain	1 (2.1%)	1/1 resolved with programming
Salmon [53]	2020	38	Revision due to lead migration	0 (0.0%)	-
			Insufficient pain relief	6 (15.8%)	6/6 had additional epidural leads placed
			IPG site pain	6 (15.8%)	6/6 managed conservatively
			Electrode high impedance	1 (2.6%)	1/1 lead replaced
			Explant due to infection	0 (0.0%)	-
Ghosh et al. [46]	2020	28	Bleeding	0 (0.0%)	-
			Infection	0 (0.0%)	-
			Neurological deficit	0 (0.0%)	-
El Majdoub et al. [50]	2019	23	Infection	3 (13.0%)	3/3 systems explanted
			Lead migration	1 (4.3%)	1/1 lead revised and pain decreased to previous level
Al-Kaisy et al. [60]	2015	15	Infection during trial	1 (6.7%)	1/1 lead(s) removed and permanent implantation planned 6 months later
		11	IPG site pain	3 (27.3%)	3/3 transient only
			Lead migration	2 (18.2%)	2/2 had lead revisions: 1/2 (post-fall) regained pain relief; 1/2 failed to respond after revision
			Neurological deficit	0 (0.0%)	-
Gill et al. [52]	2019	12	Any complications	0 (0.0%)	-

*, incidence is relative to number of implants. IPG: Implantable Pulse Generator; SCS: Spinal Cord Stimulation.

**Table 4:** Incidence of SCS complications in the literature.

Reference	Infection	IPG Site Pain	Migration	Lead Fracture
Cameron 2004 [61]	3.4%	0.9%	13.2%	9.1%
Turner et al. 2004 [62]	4.6%	5.8%	23.1%	10.2%
Kumar et al. 2006 [63]	3.4%	1.2%	21.5%	5.9%
Mekhail et al. 2011 [64]	4.5%	12.0%	22.6%	6.0%
Eldabe et al. 2015 [65]	4.9%	6.2%	15.5%	6.4%
Kleiber et al. 2016 [66]	4.2%	6.4%	0.0%	3.8%
Hoelzer et al. 2017 [67]	2.5%	-	-	-

IPG: Implantable Pulse Generator.
